# Supplementary material for: Cloud BioLinux: pre-configured and on-demand bioinformatics computing for the genomics community
Source: BMC Bioinformatics. 2012 Mar 19;13:42. doi: 10.1186/1471-2105-13-42 (PMC3372431; doi:10.1186/1471-2105-13-42)
Supplement: Additional file 1 — Supplementary 1 Cloud BioLinux software documentation in the form of a mini, self-contained website. Users need to download and uncompress the .zip file, and open through a web browser the "index.html" file available on the main directory. (ZIP 1823 kb). [file 1471-2105-13-42-S1.ZIP › Cloud-BioLinux-Package-Documentation/docs/fasty.html]

Bio-Linux Software Documentation Pages

Back to search form

## fasty

|  |  |
| --- | --- |
| Name | fasty |
| Description | **fasty** is part of the *Fasta3* package, which contains many programs for searching DNA and protein databases and for evaluating statistical significance from randomly shuffled sequences.  **fasty** compares a DNA sequence to a protein sequence data bank by comparing the translated DNA sequence in three frames and allowing gaps and frameshifts. **fasty** is slower than fastx because frameshifts are allowed within codons, but it produces better alignments with poor quality sequences. See also fastx, tfastx and tfasty.   In the Bio-Linux package, the threaded versions of the **fasta** programs are the default.   The programs available in the *Fasta3* package are:   - fasta - scan a protein or DNA sequence library for similar sequences. - fastx - compare a DNA sequence to a protein sequence database, comparing the translated DNA sequence in forward and reverse frames. - tfastx - compare a protein sequence to a DNA sequence database, calculating similarities with frameshifts to the forward and reverse orientations. - fasty - compare a DNA sequence to a protein sequence database, comparing the translated DNA sequence in forward and reverse frames.- tfasty - compare a protein sequence to a DNA sequence database, calculating similarities with frameshifts to the forward and reverse orientations.   - fasts - compare unordered peptides to a protein sequence database- tfasts - compare unordered peptides to a translated DNA sequence database- fastm - compare ordered peptides (or short DNA sequences) to a protein (DNA) sequence database- fastm - compare ordered peptides (or short DNA sequences) to a translated DNA sequence database- fastf - compare mixed peptides to a protein sequence database- tfastf - compare mixed peptides to a translated DNA sequence database             - ssearch - compare a protein or DNA sequence to a sequence database using the Smith-Waterman algorithm.             - ggsearch - compare a protein or DNA sequence to a sequence database using a global alignment (Needleman-Wunsch)- lalign - produce multiple non-overlapping alignments for protein and DNA sequences using the Huang and Miller SIM algorithm for the Waterman-Eggert algorithm. This version of **lalign** replaces that from the Fasta2 package.               - **prss** - (discontinued, replaced in the fasta35 release by new versions of ssearch and fastx) estimate statistical significance of an alignment by comparing the score to the distribution of similarity scores generated by shuffling the second sequence. **prss35** uses Smith-Waterman. **prfx35** uses the **fastx** algorithm.   **References:**  Pearson, W.R. Flexible sequence similarity searching with the FASTA3 program package. Methods Mol Biol. 2000;132:185-219 [Entrez]    Pearson, W.R. Empirical statistical estimates for sequence similarity searches. J Mol Biol. 1998 Feb 13;276(1):71-84 [Entrez]    Pearson WR, Wood T, Zhang Z, Miller W. Comparison of DNA sequences with protein sequences. Genomics. 1997 Nov 15;46(1):24-36. [Entrez] |
| Homepage | http://www.people.virginia.edu/~wrp/pearson.html |
| Remote Documentation | http://www.people.virginia.edu/~wrp/papers/ismb2000.pdf |
